# Patient-Reported Outcomes on Quality of Life in Older Adults with Oral Pemphigus

**DOI:** 10.3390/healthcare13222843

**Published:** 2025-11-09

**Authors:** Emily-Alice Russu, Liliana Gabriela Popa, Stana Păunică, Lucia Bubulac, Călin Giurcăneanu, Cristina-Crenguța Albu

**Affiliations:** 1 Doctoral School, “Carol Davila” University of Medicine and Pharmacy, 020021 Bucharest, Romania; emily-alice.russu@drd.umfcd.ro; 2 Department of Oncologic Dermatology, Elias Emergency University Hospital, “Carol Davila” University of Medicine and Pharmacy, 020021 Bucharest, Romania; liliana.popa@umfcd.ro (L.G.P.); calin.giurcaneanu@umfcd.ro (C.G.); 3 Department of Periodontology, Faculty of Dentistry, “Carol Davila” University of Medicine and Pharmacy, 020021 Bucharest, Romania; 4 Department of Family Medicine, Faculty of Medicine, “Carol Davila” University of Medicine and Pharmacy, 020021 Bucharest, Romania; 5 Department of Genetics, Faculty of Dentistry, “Carol Davila” University of Medicine and Pharmacy, 020021 Bucharest, Romania; cristina.albu@umfcd.ro

**Keywords:** oral pemphigus, pemphigus vulgaris, patient-reported outcomes, quality of life, OP-QoLQ, DLQI, ABSIS, older adults

## Abstract

**Background**: Oral pemphigus is a rare autoimmune blistering disorder predominantly affecting the mucous membranes, particularly in older adults. Despite therapeutic advances, the chronic, painful, and recurrent nature of oral pemphigus vulgaris substantially impairs patients’ quality of life (QoL). Patient-reported outcomes (PROs) offer valuable insights into the subjective burden of the disease; however, data on PROs in older adults with oral pemphigus are scarce. Objective: To assess QoL in older adults diagnosed with oral pemphigus using validated PRO measures and to identify key clinical factors associated with QoL deterioration. **Methods**: A cross-sectional pilot study was conducted involving 10 participants aged 60 years or older with confirmed oral pemphigus vulgaris. Participants completed the Oral Pemphigus–Specific Quality of Life Questionnaire (OP-QoLQ) and the Dermatology Life Quality Index (DLQI). Clinical severity was evaluated using the Autoimmune Bullous Skin Disorder Intensity Score (ABSIS). Statistical analyses explored correlations between disease severity, treatment regimens, and QoL outcomes. **Results**: Most participants reported moderate to severe QoL impairment, with eating difficulties and emotional distress being the most frequently mentioned issues. Higher ABSISs and longer disease duration were significantly correlated with poorer OP-QoLQ and DLQI outcomes (Spearman’s ρ up to 0.80; *p* ≤ 0.021). Systemic corticosteroid therapy was more frequently reported among those with advanced disease, although treatment-related adverse effects may contribute to reduced QoL. **Conclusion**: Oral pemphigus substantially compromises QoL in older adults, with both disease- and treatment-related factors playing important roles. These findings support the integration of PROs into the multidisciplinary management of older adults with oral pemphigus vulgaris.

## 1. Introduction

Oral health is a cornerstone of general well-being, particularly in older adults, where the ability to eat comfortably, speak clearly, and engage socially is essential for maintaining independence and quality of life. Chronic oral mucosal diseases can compromise these vital functions but are often underrecognized and undertreated in geriatric care settings [1]. Among these conditions, pemphigus vulgaris (PV) occupies a distinctive position as a rare, autoimmune blistering disorder primarily affecting the oral mucosa. It is characterized by the formation of fragile intraepithelial blisters that rupture easily, leading to painful oral erosions [2]. In older adults, the disease burden is magnified by immunosenescence, multimorbidity, and polypharmacy, which together complicate both diagnosis and management [3].

### 1.1. Epidemiology and Burden of Oral Pemphigus in Older Adults

Oral pemphigus, most frequently manifesting as the mucosal-dominant form of PV, is a rare but highly disabling autoimmune blistering disease of the oral cavity. Epidemiological studies estimate an annual incidence of approximately 0.1–0.5 cases per 100,000 person-years, with variation influenced by ethnicity, geographic region, and genetic predisposition [4,5]. Although PV can occur at any age, older adults represent a distinct subgroup in which the disease is often more severe, chronic, and therapeutically challenging [6]. In these patients, diagnosis may be delayed due to overlapping presentations with other oral mucosal conditions, such as lichen planus, aphthous stomatitis, or traumatic mucosal ulcerations [7,8,9].

Chronic pain, masticatory impairment, and bleeding lesions frequently result in altered dietary intake, malnutrition, and weight loss [10]. When combined with pre-existing comorbidities—such as diabetes, cardiovascular disease, or osteoporosis—these complications accelerate systemic decline, increased frailty, and loss of independence [11]. The burden of PV in older populations is therefore both medical and socioeconomic, demanding integrated, multidisciplinary approaches that address oral and systemic health in tandem.

### 1.2. Pathophysiology and Age-Associated Vulnerabilities

PV is mediated by pathogenic IgG autoantibodies targeting desmosomal cadherins—primarily desmoglein 3 (Dsg3) and, in some cases, desmoglein 1 (Dsg1)—leading to disruption of keratinocyte adhesion (acantholysis) and intraepithelial blister formation [12]. In the oral mucosa, the predominance of Dsg3 expression explains why oral erosions often precede cutaneous lesions in mucosal-predominant PV [13]. Older adults exhibit unique pathophysiological vulnerabilities. Immunosenescence is characterized by diminished adaptive immunity and dysregulated inflammatory signaling, that modulate disease expression and delay healing [14]. Age-related decline in mucosal regenerative capacity prolongs ulcer persistence, while polypharmacy raises the risk of drug interactions and treatment-related adverse effects [5,6,14,15,16]. Long-term systemic corticosteroid therapy—the standard first-line treatment—carries heightened risks in this age group, including osteoporosis, impaired glucose tolerance, hypertension, and increased susceptibility to infection [17]. Alternative therapies, such as rituximab, have shown promise in reducing steroid dependence [18], but strong safety and efficacy data for geriatric patients remain limited. Consequently, treatment decisions in elderly PV must balance disease suppression with preservation of systemic health.

### 1.3. Oral Health–Related Quality of Life in Geriatric Populations

Oral health–related quality of life (OHRQoL) is a multidimensional tool describing the impact of oral health on functional ability, psychological well-being, and social participation [19]. In older populations, maintaining high OHRQoL is essential for preserving nutritional status, speech, and social integration [20]. Poor OHRQoL has been linked to depression, cognitive decline, and shortened life expectancy [21].

Evidence from geriatric dentistry literature reinforces the connection between oral disease and diminished QoL [22]. Recent studies have shown that xerostomia and impaired masticatory function markedly reduced OHRQoL in rural-dwelling older adults [23,24,25]. Fouda et al. reported that prosthetic rehabilitation improved both functional ability and psychosocial well-being in edentulous older adult patients [26]. Given the similar functional impairments caused by PV—persistent pain, difficulty chewing, and dietary restriction—these findings are directly relevant, yet remain underexplored in the context of autoimmune mucosal disease. In oral pemphigus, these OHRQoL determinants become even more pronounced because chronic mucosal erosions continuously impair oral function, reduce tolerance to nutritional intake, and significantly accelerate the decline in quality of life in older adults.

### 1.4. Patient-Reported Outcomes in Autoimmune Oral Diseases

Patient-reported outcomes (PROs) provide direct insights into patients’ perceptions of symptoms, functional status, and treatment effects, complementing clinician-reported and objective measures [27]. In autoimmune blistering diseases, PROs reveal specific domains of disease burden—such as fatigue, social withdrawal, or emotional distress—that are invisible to lesion counts or severity indices [28]. ABSIS is a validated clinical severity tool, but it does not account for the subjective dimensions of disease impact [29].

Combining PROs such as OP-QoLQ and DLQI with ABSIS can provide a holistic understanding of PV’s impact, guiding individualized treatment strategies. Despite this, Askin et al. observed that most PV studies do not stratify PRO findings by age, leaving a major knowledge gap regarding the specific needs of older adults [30]. This is particularly important because older adults may prioritize comfort, independence, and function over complete lesion clearance.

### 1.5. Research Gap and Rationale for the Present Study

Despite recognition of PV as a disease with profound functional and psychosocial consequences, its specific impact on older adults with predominant oral involvement remains poorly defined. No targeted studies have examined the relationship between disease severity (ABSIS) and OHRQoL (OP-QoLQ, DLQI) in older adults. Moreover, there is no standardized framework for incorporating PROs into the routine management of PV in this population segment.

*Novelty statement:* To our knowledge, this is the first study to concurrently assess clinical severity and validated OHRQoL measures in an exclusively older adult PV cohort. By integrating clinical and patient-reported outcomes, we provide evidence that not only quantifies the burden of oral PV in older adults but also offers actionable insights for patient-centered, multidisciplinary care.

## 2. Materials and Methods

Each methodological step was selected to align with the primary objective of this study: to quantify oral disease severity in older adults with PV and evaluate its association with patient-reported quality of life.

### 2.1. Study Design and Ethical Approval

This observational, cross-sectional pilot study was conducted between May 2024 and September 2025 at the Department of Oncologic Dermatology, Elias Emergency University Hospital, Bucharest, Romania. The study aimed to evaluate OHRQoL and dermatology-specific quality of life in older adults (≥60 years) diagnosed with oral PV, and to examine their association with clinically assessed disease severity.

The study protocol adhered to the ethical principles of the Declaration of Helsinki (2013 revision) and received final approval from the Institutional Ethics Council of Elias Emergency University Hospital, Bucharest, Romania (Approval No. 4180/18.07.2025), as well as from the Scientific Research Ethics Commission of the “Carol Davila” University of Medicine and Pharmacy, Bucharest, Romania (Approval No. 24375/23.09.2025).

All participants provided written informed consent after receiving detailed information regarding study aims, procedures, potential risks, and their right to withdraw at any stage without affecting their ongoing medical care. Confidentiality was ensured by coding all participant data and restricting access to authorized investigators, with only aggregated results being reported to prevent individual identification. Participation was entirely voluntary, with no financial incentives offered.

**Figure 1:**
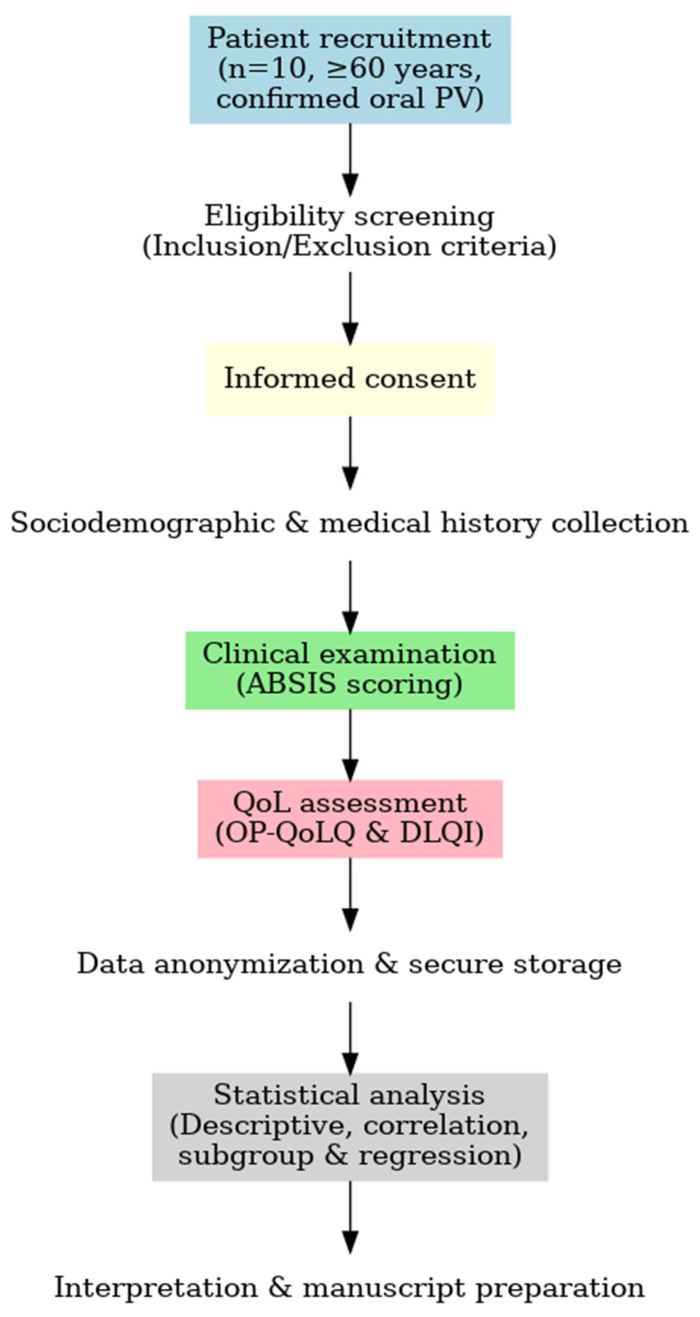
Workflow of the study assessing the relationship between disease severity and quality of life in older adults with oral pemphigus vulgaris (*n* = 10). During eligibility screening, 2 patients were excluded due to severe cognitive impairment that prevented valid questionnaire completion.

Figure 1 illustrates the sequential workflow of the study, from patient recruitment to data analysis and manuscript preparation.

### 2.2. Participants

#### 2.2.1. Inclusion Criteria

Participants were eligible if they met all the following:

- Age ≥ 60 years at recruitment.
- Confirmed diagnosis of oral PV based on clinical examination showing oral mucosal erosions/blisters, histopathology demonstrating intraepithelial blister formation with acantholysis, and direct immunofluorescence (DIF) revealing intercellular IgG and/or C3 deposition.
- Predominant oral involvement (with or without limited skin lesions) within the last six months.
- Ability to complete questionnaires independently or with minimal assistance.

#### 2.2.2. Exclusion Criteria

Exclusion criteria included:

- Other autoimmune mucocutaneous disorders (e.g., mucous membrane pemphigoid, erosive lichen planus).
- Severe cognitive or psychiatric impairment preventing valid questionnaire completion.
- Acute systemic illness or hospitalization within the past four weeks.
- Participation in another interventional clinical trial at the time of recruitment.

#### 2.2.3. Sample Size Justification

The study cohort was intentionally limited due to the rarity of oral pemphigus vulgaris in the older adult population and the exploratory nature of the research. Although regression-based statistical models typically require a minimum of 98 participants (calculated using the formula N ≥ 50 + 8 m, where m = number of independent predictors), the recruitment of such a sample size was not feasible in the context of a low-incidence disease. Consequently, the present investigation was designed as a pilot study, involving 10–15 participants, each of whom completed two validated patient-reported outcome measures (OP-QoLQ and DLQI). This approach generated a total of 20–30 completed questionnaires, providing paired data for clinical and quality-of-life analyses. The limited sample size reflected the strict inclusion criteria, the advanced age of participants, and the need to ensure comprehensive clinical and patient-reported assessments within a single visit. The primary objective was to generate preliminary, age-specific data on the relationship between clinical severity and quality of life in older adults with oral PV. These findings are intended to inform the development of larger, multicenter investigations with sufficient statistical power to validate and expand upon the observed trends.

### 2.3. Clinical Evaluation

All participants underwent a standardized oral examination performed by a calibrated examiner. Disease severity was quantified using the Autoimmune Bullous Skin Disorder Intensity Score (ABSIS), which incorporates lesion extent and patient-reported discomfort during eating/drinking. The oral subscore (range: 0–11) considered both the number of affected oral sites and the severity of pain.

Calibration was performed prior to study initiation, with intra-rater reliability assessed in a pilot group (intraclass correlation coefficient > 0.90).

### 2.4. Patient-Reported Outcome Measures

The assessment of PROs was central to this study’s design, reflecting the multidimensional impact of oral PV on functional, psychological, and social domains. Two complementary instruments were selected: the Oral Pemphigus–Specific Quality of Life Questionnaire (OP-QoLQ), capturing disease-specific burdens, and the Dermatology Life Quality Index (DLQI), enabling comparison with broader dermatology and mucosal disease cohorts. This dual approach ensured that both condition-specific and general health-related quality of life (QoL) dimensions were addressed.

#### 2.4.1. Oral Pemphigus–Specific Quality of Life Questionnaire (OP-QoLQ)

The OP-QoLQ is a condition-specific tool developed to measure the impact of oral pemphigus on daily life. Details of the OP-QoLQ items and scoring are provided in the Supplementary Materials (Table S1). It was adapted from validated oral mucosal disease QoL instruments to ensure content relevance for autoimmune blistering disorders. The questionnaire includes 15 items distributed across five domains:

1. Physical symptoms—pain, burning, bleeding, difficulty swallowing.
2. Functional limitations—chewing, speaking, maintaining oral hygiene.
3. Emotional well-being—anxiety, embarrassment, frustration.
4. Social participation—avoidance of social gatherings, communication difficulties.
5. Treatment burden—side effects, time for care, treatment accessibility.

Each item is scored on a 5-point Likert scale (0 = “never” to 4 = “very often”), yielding a total score between 0 and 60. Higher scores indicate greater QoL impairment. For interpretability, scores were classified as mild (0–20), moderate (21–40), and severe (41–60) impact.

The recall period for all items was standardized to the preceding two weeks, ensuring comparability with DLQI data and reducing recall bias. Minor linguistic adjustments were made to improve comprehension among older adults, and the adapted version was pretested in a pilot group of older adults with oral PV to ensure clarity and feasibility. No formal psychometric revalidation was performed, but the structure and scoring of the original instrument were preserved.

#### 2.4.2. Dermatology Life Quality Index (DLQI)

The DLQI is a validated 10-item instrument assessing the impact of skin and mucosal diseases on QoL over the preceding two weeks. It covers six domains:

1. Symptoms and feelings
2. Daily activities
3. Leisure
4. Work and school
5. Personal relationships
6. Treatment

Each item is scored from 0 (“not at all”) to 3 (“very much”), for a total of 0–30 points. The standard interpretation is:

- 0–1: no effect
- 2–5: small effect
- 6–10: moderate effect
- 11–20: very large effect
- 21–30: extremely large effect

For this study, the DLQI was adapted to include oral-specific symptom prompts, while retaining its original structure and scoring to maintain comparability. The adapted version was pretested in the target older adult population to confirm clarity and cultural relevance; no formal revalidation was undertaken.

Table 1 summarizes the structure, scoring, and sources of both instruments.

Both questionnaires were administered in a private setting. Assistance was available for visual or motor impairments, without influencing responses.

#### 2.4.3. Rationale for Instrument Selection

The OP-QoLQ was chosen for its sensitivity to oral disease-specific functional limitations and treatment burdens, while the DLQI was included to enable benchmarking with a broad range of dermatologic and mucosal conditions. Together, these tools provided a complementary and multidimensional assessment of QoL, which is particularly important in older adults given the interplay between oral function, systemic health, and psychosocial well-being. Although OP-QoLQ and DLQI have been previously validated in oral mucosal and dermatologic populations, their psychometric performance in older cohorts has not yet been specifically established. This pilot study aims to contribute preliminary evidence in this direction.

### 2.5. Data Collection Procedure

Following the selection of PRO instruments, participants attended a single study visit. After obtaining informed consent, the following were recorded:

- Sociodemographic data—age, sex, education level, living situation.
- Medical history—disease duration, comorbidities, current treatments.
- Clinical assessment—ABSIS scoring by the calibrated examiner.
- Patient-reported outcomes—self-administration of OP-QoLQ and DLQI in a quiet, private setting. Assistance was available for participants with visual or motor limitations, without influencing responses.

Data were anonymized using coded identifiers and stored in a secure database accessible only to study investigators.

### 2.6. Statistical Analysis

All statistical analyses were performed using IBM SPSS Statistics for Windows, Version 26.0 (IBM Corp., Armonk, NY, USA). A *p*-value < 0.05 (two-tailed) was considered statistically significant. Given the rarity of oral pemphigus vulgaris in the older adult population and the exploratory, pilot nature of this study, descriptive and bivariate analyses were prioritized over confirmatory multivariate modeling.

Descriptive statistics were used to summarize sociodemographic, clinical, and patient-reported outcome variables. Continuous variables were expressed as mean ± standard deviation (SD) or median (interquartile range, IQR) depending on the distribution assessed with the Shapiro–Wilk test. Categorical variables were presented as frequencies and percentages. Missing data were handled by listwise deletion in bivariate analyses.

For the primary analysis, correlations between clinical severity (ABSIS total and oral subscores) and quality of life measures (OP-QoLQ and DLQI total scores) were evaluated using Pearson’s correlation coefficient for normally distributed data and Spearman’s rank correlation coefficient for non-normally distributed data.

Secondary analyses included:

- Subgroup comparisons—Independent-samples t-tests or Mann–Whitney U tests were applied to compare QoL scores by sex, comorbidity status, and disease duration (<2 years vs. ≥2 years).
- Multiple group comparisons—One-way ANOVA or Kruskal–Wallis tests were used to explore differences in QoL across treatment groups (systemic corticosteroids, rituximab, combination therapy).

Exploratory multivariate analyses (multiple linear regression) were conducted to identify potential predictors of QoL impairment, with OP-QoLQ and DLQI total scores as dependent variables. Independent variables included age, sex, disease duration, ABSIS, comorbidity status, and treatment type. Given the limited sample size, these regression models were considered hypothesis-generating rather than confirmatory, and their results were interpreted with caution. Variance inflation factors (VIF) were examined to assess multicollinearity, and model fit was reported using adjusted R^2^ values.

Given the inherently small sample size related to the low prevalence of oral pemphigus vulgaris and the strict inclusion criteria, the study was not powered to detect small effect sizes. The analyses were intended to identify clinically relevant trends and generate preliminary effect size estimates to inform sample size calculations for future multicenter research. Effect sizes (ρ or r) and 95% confidence intervals (CIs) were calculated using Fisher’s z-transformation. Effect size interpretation followed Cohen’s thresholds (small < 0.3, moderate 0.3–0.5, large > 0.5). To minimize selection and reporting bias, all participants were consecutively recruited and completed the questionnaires in a standardized setting, with no proxy responses or caregiver mediation. No formal sensitivity analysis was performed due to the intentionally limited sample size and the exploratory design of the study.

## 3. Results

### 3.1. Participant Characteristics

A total of 10 older adults (aged 60 years or older) with confirmed oral pemphigus vulgaris were included in the study. The mean age of the group was 68.4 ± 5.9 years (range, 60–78 years), with female predominance (*n* = 6, 60%). The median disease duration was 3.2 years (interquartile range [IQR], 1.8–5.4 years). Eight participants (80%) reported at least one chronic comorbidity, most frequently hypertension (*n* = 6, 60%) and type 2 diabetes mellitus (*n* = 3, 30%). At the time of assessment, systemic corticosteroids represented the most common treatment modality (*n* = 7, 70%), followed by rituximab monotherapy (*n* = 2, 20%) and combined corticosteroid–rituximab therapy (*n* = 1, 10%). Table 2 summarizes the demographic, clinical, and treatment characteristics of the study population.

### 3.2. Patient-Reported Outcomes

The mean OP-QoLQ total score was 38.6 ± 8.9, reflecting a moderate-to-severe quality-of-life (QoL) impact. The DLQI mean total score was 14.2 ± 4.5, corresponding to a substantial effect on daily life according to the instrument’s interpretation thresholds.

Internal consistency and item–total patterns were examined qualitatively; formal reliability estimates are deferred to larger studies. As hypothesized, OP-QoLQ totals correlated strongly with disease severity (ABSIS total/oral subscore) and with DLQI (ρ ~0.70–0.80; *p* < 0.05), supporting convergent validity in this exploratory sample.

Within the OP-QoLQ, functional limitations and physical symptoms were the most affected domains. Difficulty eating hard foods was reported as “often” or “very often” by 80% of patients, and 90% experienced persistent oral pain or burning. In the DLQI, the most impacted domains were symptoms and feelings, as well as daily activities.

**Figure 2:**
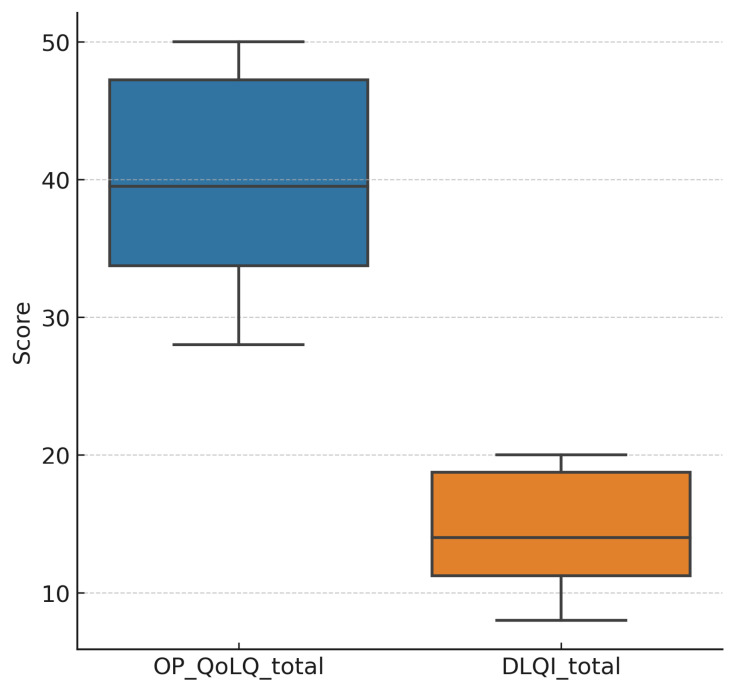
Distribution of OP-QoLQ and DLQI total scores in older adults with oral pemphigus vulgaris. Legend: Boxplots display the median (horizontal line) and interquartile range (IQR; box). Whiskers represent the minimum and maximum observed values within 1.5 × IQR. Higher scores indicate worse quality of life. Both measures revealed substantial impairment, with OP-QoLQ showing a wider distribution range, reflecting heterogeneity in oral symptom burden.

Figure 2 shows the distribution of OP-QoLQ and DLQI scores, highlighting substantial interindividual variability, yet consistently elevated impairment levels.

### 3.3. Correlation Between Disease Severity and Quality of Life

The ABSIS total scores ranged from 9 to 48 (mean: 27.4 ± 10.5), with higher values indicating greater clinical severity. Strong, statistically significant positive correlations were observed between ABSIS and QoL measures (two-tailed Spearman’s ρ):

- ABSIS total vs. OP-QoLQ: ρ = 0.78, *p* = 0.008
- ABSIS total vs. DLQI: ρ = 0.71, *p* = 0.021
- ABSIS oral subscore vs. OP-QoLQ: ρ = 0.80, *p* = 0.006
- ABSIS oral subscore vs. DLQI: ρ = 0.74, *p* = 0.015

These findings indicate that higher levels of clinical disease activity were associated with greater patient-reported impairment across both disease-specific and general dermatology-related QoL instruments. The associations were slightly stronger for the oral-specific OP-QoLQ compared to the dermatology-specific DLQI.

**Figure 3:**
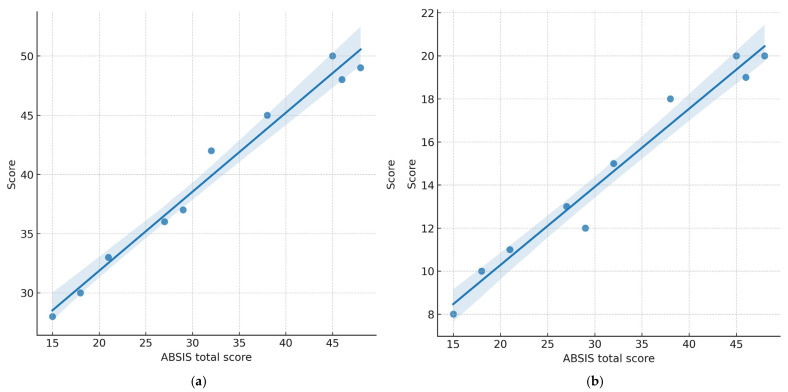
Correlations between clinical severity (ABSIS total score) and quality of life (QoL) in older adults with oral pemphigus vulgaris (*n* = 10). (**a**) ABSIS vs. OP-QoLQ total score (Spearman’s ρ = 0.78; *p* = 0.008); (**b**) ABSIS vs. DLQI total score (Spearman’s ρ = 0.71; *p* = 0.021). Each point represents one participant. Lines depict fitted linear regression; shaded areas indicate 95% confidence intervals. Higher ABSISs were associated with worse QoL, with a stronger association for the oral-specific OP-QoLQ. Note: Spearman’s rho (ρ) was used as the correlation coefficient due to the small sample size and non-parametric data distribution.

Figure 3 illustrates the associations between clinical severity (ABSISs) and patient-reported quality of life.

### 3.4. Subgroup Analyses

Subgroup comparisons provided additional insights (two-tailed *p*-values reported):

- Sex: No statistically significant differences in OP-QoLQ (*p* = 0.42) or DLQI (*p* = 0.38) between males and females.
- Comorbidity status: Participants with one or more comorbidities had significantly higher OP-QoLQ scores (41.5 ± 7.6) compared to those without comorbidities (28.0 ± 4.2, *p* = 0.041).
- Disease duration: Those with disease duration of ≥2 years had significantly higher OP-QoLQ scores (42.3 ± 6.5) than patients with disease duration of <2 years (31.4 ± 5.9, *p* = 0.038). DLQI showed a similar trend (*p* = 0.049).
- Treatment type: The highest impairment was observed in the combination therapy group, followed by corticosteroids alone. Patients receiving rituximab monotherapy had comparatively lower mean scores, although these differences were not statistically significant (*p* > 0.05).

**Figure 4:**
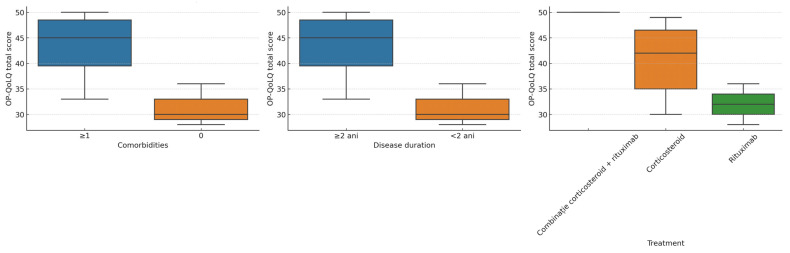
Boxplots of OP-QoLQ scores stratified by comorbidity status, disease duration, and treatment type. Legend: Boxplots display the median (horizontal line) and IQR (box); whiskers show the range within 1.5 × IQR. Patients with ≥1 comorbidity and those with a disease duration ≥ 2 years had higher OP-QoLQ scores, indicating greater impairment. Differences by treatment type were less pronounced.

Figure 4 illustrates these subgroup differences.

### 3.5. Exploratory Regression Analyses

Multiple linear regression models identified ABSIS total score and disease duration as the strongest predictors of QoL impairment:

- OP-QoLQ model: Adjusted R^2^ = 0.62, *p* < 0.01
- DLQI model: Adjusted R^2^ = 0.58, *p* < 0.01

Neither age nor sex emerged as a significant predictor. Variance inflation factors (VIF < 2) confirmed minimal multicollinearity.

Table 3 presents detailed regression coefficients.

## 4. Discussion

### 4.1. Principal Findings

In this pilot cohort of older adults with oral PV, higher clinical severity—captured by the Autoimmune Bullous Skin Disorder Intensity Score (ABSIS) total and oral subscores—and longer disease duration were independently associated with poorer quality of life (QoL), as assessed using both an oral-specific instrument (OP-QoLQ) and a dermatology-generic instrument (DLQI). Effect sizes were large (Spearman’s ρ = 0.71–0.80), and exploratory regression models indicated that ABSIS and disease chronicity together explained more than half of the variance in QoL outcomes. In contrast, demographic variables such as age and sex were not significant predictors.

Subgroup analyses suggested that a higher comorbidity burden and longer disease duration were linked to worse OP-QoLQ scores, with a non-significant trend toward better QoL among patients treated with rituximab compared with those receiving corticosteroids alone or in combination therapy. These findings underscore the central role of mucosal disease activity and the cumulative burden of disease duration in shaping patient-reported outcomes in the geriatric oral PV population.

### 4.2. Comparison with Existing Literature

Our results are consistent with contemporary evidence showing that disease activity in pemphigus closely parallels declines in health-related quality of life (HRQoL) [33]. Recent outcome research in autoimmune blistering diseases (AIBDs) has confirmed that validated severity indices—such as the Autoimmune Bullous Skin Disorder Intensity Score (ABSIS) and the Pemphigus Disease Area Index (PDAI)—correlate strongly with patient-reported outcome (PRO) measures, including the Dermatology Life Quality Index (DLQI), the Autoimmune Bullous Disease Quality of Life (ABQOL) questionnaire, and disease-specific tools [34,35,36]. The inclusion of both a clinical severity index and a PRO in our study mirrors these recommendations and provides converging evidence for the strong ABSIS–QoL associations found in our cohort.

Mucosal involvement—especially oral lesions—has been repeatedly identified as a key determinant of HRQoL in pemphigus [33,37]. Alshami et al. (2021) conducted a 20-year retrospective analysis of oral PV cases and found that most patients, predominantly females, experienced severe pain and functional limitation despite ongoing treatment, significantly affecting nutrition and social interaction [38]. These results parallel our observation that even in treated elderly patients, oral disease activity remains a major driver of impairment. Similarly, Davis et al. (2024) reported that uncontrolled intraoral lesions have direct and profound effects on eating ability, oral hygiene maintenance, and psychosocial well-being, reinforcing the central role of oral lesion control in improving patient-reported outcomes [39].

Our findings also align with those of Bilgiç et al. (2020), who examined OHIP-14 and ABSISs in a cross-sectional AIBD cohort and demonstrated significantly higher OHIP-14 scores among patients with active disease (ABSIS ≥ 17) compared to those with lower disease activity [40]. This parallels our data, where greater ABSISs—particularly the oral subscore—were strongly associated with worse OP-QoLQ and DLQI results.

Broader oral medicine literature confirms that chronic oral mucosal diseases lead to sustained QoL impairment [41]. Kajla et al. (2024) reported that pain and functional limitation are the primary determinants of poor oral health-related QoL across a range of mucosal conditions [42]. While that study assessed a heterogeneous patient population, the pattern is highly consistent with our OP-QoLQ findings in oral PV, underscoring that oral-specific PRO instruments offer greater granularity for functional and symptom-related impacts than generic dermatology measures [43].

From a global perspective, multicenter registries such as the European Reference Network for Rare and Complex Skin Diseases (ERN-Skin) have reported similar trends, with mucosal-dominant PV phenotypes showing disproportionately high HRQoL burden compared with cutaneous-dominant forms [44]. Notably, the prevalence of oral-predominant disease is higher in Southern European and Mediterranean populations [45], which may partly explain the persistent QoL impairment in our Romanian elderly cohort despite treatment.

Treatment-related trends in our data—suggesting better QoL outcomes in rituximab-treated patients—are directionally consistent with recent evidence from observational cohorts and clinical trials. Multiple studies have shown significant DLQI improvements within 6–12 months after rituximab initiation, alongside reductions in corticosteroid requirements and flare frequency [46,47,48]. Although our sample was underpowered for statistical confirmation, the observed pattern supports the hypothesis that early, targeted B-cell depletion may yield tangible QoL benefits, especially in elderly patients at risk of steroid-related toxicity.

Taken together, these findings extend the current literature by focusing specifically on an older, predominantly oral PV population—an underrepresented group in pemphigus research—and by confirming that both disease severity and chronicity are robust predictors of QoL impairment across complementary measurement tools [13,49,50]. This reinforces the concept that in oral PV, controlling mucosal disease activity is not only a clinical imperative but also a key determinant of patient-perceived well-being.

### 4.3. Potential Biological and Clinical Explanations

The strong association between ABSIS oral scores and QoL is biologically and clinically plausible. The oral mucosa is frequently the earliest and most persistent site of PV activity, a pattern attributable to the high density of desmoglein-3 expression and the distinct mechanical, microbial, and chemical environment of the oral cavity [51]. Persistent erosions interfere mastication, speech, and oral hygiene, leading to physical discomfort, nutritional compromise, and social withdrawal—domains particularly well captured by the OP-QoLQ instrument [52].

Disease chronicity amplifies these effects through cumulative mucosal damage, repeated flares, and prolonged exposure to systemic corticosteroids, which carry significant metabolic, musculoskeletal, and psychological side effects [53]. Aging-related immune changes (immunosenescence) reduce tissue regenerative capacity, while multimorbidity and polypharmacy in older adults may slow mucosal healing and heighten pain perception [54]. Cardiovascular and metabolic comorbidities can impair wound healing and amplify inflammatory pain, providing a plausible explanation for the observed association between greater comorbidity burden and lower OP-QoLQ scores [16,55,56].

At the immunopathological level, PV pathogenesis is driven by pathogenic IgG autoantibodies targeting desmoglein-1 and desmoglein-3, leading to acantholysis and blister formation [13,57]. B-cell depletion with rituximab reduces autoantibody production, promotes mucosal healing, and decreases corticosteroid dependence [18]. Observational cohorts and real-world studies consistently report significant DLQI and oral health–related QoL improvements following rituximab, with maximal gains within 6–12 months [58,59]. Although our study lacked statistical power to confirm this effect, the observed trend toward improved QoL among rituximab-treated patients supports its potential therapeutic role in elderly oral PV, particularly when introduced early within steroid-sparing regimens.

### 4.4. Strengths and Unique Contribution

This study offers several notable strengths that address a clear evidence gap in the pemphigus literature. It is among the few investigations to focus exclusively on older adults with oral PV—a demographic whose clinical course and quality-of-life concerns are often underrepresented in research. By integrating a clinician-rated disease severity index (ABSIS) with both an oral-specific (OP-QoLQ) and a dermatology-generic (DLQI) instrument, the analysis captures both the objective burden of mucosal disease and its subjective functional impact with greater granularity than generic tools alone. Furthermore, the identification of disease duration and comorbidity burden as independent predictors of QoL underlines geriatric-specific vulnerabilities that have received limited attention in prior studies. Collectively, these findings provide a coherent and clinically meaningful framework to guide patient-centered monitoring and inform the design of future interventional trials aimed at improving outcomes in this high-risk population.

### 4.5. Implications for Clinical Practice and Health Policy

The present findings emphasize the clinical relevance of embedding structured patient-reported outcome (PRO) assessments into the routine management of older adults with oral PV. In busy clinical environments, combining ABSIS, particularly its oral subscore, with a concise, validated QoL instrument provides an evaluation of objective disease burden and the patient’s subjective experience, thereby enabling timely and individualized therapeutic adjustments. The stronger correlation found for the oral-specific OP-QoLQ demonstrates that oral-focused instruments better capture functional impairments (speaking and chewing) than generic dermatology scales, which tend to underestimate oral disability.

These observations also support earlier adoption of steroid-sparing strategies, including rituximab, in elderly patients with significant mucosal involvement, given the growing body of evidence for both its efficacy and associated QoL benefits. From a health policy perspective, incorporating PRO measures into clinical quality indicators and reimbursement frameworks could foster patient-centered care while ensuring equitable and timely access to biologics and coordinated multidisciplinary oral health services for this high-burden, low-prevalence condition.

We therefore recommend that PRO instruments be systematically integrated into routine geriatric dermatology and oral medicine consultations, as a standard component of disease monitoring in oral PV.

## 5. Future Research

Future studies should prioritize multicenter, longitudinal designs enrolling at least 50–100 participants to validate these associations and map trajectories of QoL in relation to disease activity and therapeutic interventions. The integration of objective biomarkers, such as desmoglein 1 and 3 antibody titers, inflammatory cytokine profiles, and oral microbiome signatures, could bring more profound insights into the biological drivers of QoL impairment. Furthermore, advanced analytic approaches, including multi-omic integration and machine learning–based predictive modeling, may help identify patients at the highest risk of sustained QoL deterioration, enabling earlier, personalized interventions. Comparative analyses between geriatric and younger PV cohorts will be essential to elucidate age-specific determinants of HRQoL and guide the development of tailored, population-specific management strategies.

## 6. Limitations of the Study

This pilot study has several limitations. The small sample size, single-center recruitment, and cross-sectional design inherently limit generalizability and preclude causal inferences regarding treatment effects. Although the adapted OP-QoLQ demonstrated consistent performance within this cohort, formal psychometric validation in older adults with oral PV remains necessary to ensure its reliability and sensitivity in this demographic. Accordingly, OP-QoLQ findings in this study should be considered hypothesis-generating and require confirmation in larger, multicenter cohorts with full psychometric validation (e.g., factor structure, test–retest reliability).

## 7. Conclusions

To our knowledge, this is the first study focused exclusively on older adults with oral-predominant pemphigus vulgaris, demonstrating that mucosal disease severity and chronicity are strong and independent predictors of impaired quality of life. The stronger associations observed with the oral-specific OP-QoLQ confirm that generic dermatology scales systematically underestimate the true degree of functional disability in this phenotype.

Clinically, these findings support systematic integration of validated patient-reported outcome instruments into routine geriatric dermatology and oral medicine consultations. Larger multicenter studies are needed to validate these pilot results, define clinically meaningful change thresholds, and establish PRO-guided treatment algorithms in oral pemphigus vulgaris. To establish PRO-guided treatment algorithms for oral pemphigus vulgaris, define clinically meaningful change thresholds, and validate these pilot results, larger multicenter studies are necessary. In order to guide future therapeutic trials and convert these results into evidence-based, patient-centered care pathways, it will be crucial to establish standardized, age-specific PRO benchmarks.

## Figures and Tables

**Table 1:** Summary of patient-reported outcome measures used in the study.

Instrument	Domains Assessed	No. of Items	Scoring System	Interpretation	Recall Period	Adaptation & Validation	Reference
OP-QoLQ (Oral Pemphigus–Specific Quality of Life Questionnaire)	Physical symptoms, functional limitations, emotional well-being, social participation, treatment burden	15	5-point Likert (0 = never to 4 = very often); total score range: 0–60	0–20 = mild impact; 21–40 = moderate impact; 41–60 = severe impact	2 weeks	Minor linguistic adjustments to enhance comprehension among older adults; pilot pretesting conducted in older adults with oral pemphigus vulgaris to ensure cultural and linguistic appropriateness; no formal psychometric revalidation performed	López-Jornet et. al. [31]
DLQI (Dermatology Life Quality Index)	Symptoms & feelings, daily activities, leisure, work/school, personal relationships, treatment	10	4-point Likert (0 = not at all to 3 = very much); total score range: 0–30	0–1 = no effect; 2–5 = small effect; 6–10 = moderate effect; 11–20 = very large effect; 21–30 = extremely large effect	2 weeks	Inclusion of oral symptom–specific prompts to improve disease relevance; pilot pretesting for clarity and cultural appropriateness in older adult patients; no formal psychometric revalidation performed	Finlay et al. [32]

Legend: OP-QoLQ = Oral Pemphigus–Specific Quality of Life Questionnaire; DLQI = Dermatology Life Quality Index. The OP-QoLQ was selected for its sensitivity to oral function limitations and treatment-related burden specific to autoimmune blistering diseases, particularly relevant in older adult populations. Minor linguistic adaptations and pilot testing in elderly patients with oral pemphigus vulgaris ensured cultural and comprehension appropriateness without altering the instrument’s core structure. The DLQI was chosen to enable cross-condition comparisons across dermatology and oral mucosal disorders. Oral symptom–specific prompts were added to improve relevance, with pilot testing confirming clarity for the target population. No formal psychometric revalidation was performed for either instrument, as the aim was to preserve their validated structure while enhancing applicability to this specific patient group. These instruments were integral to capturing disease-specific and general quality-of-life impacts. They aligned directly with the study’s objective of comprehensively evaluating patient-reported outcomes in older adults with oral pemphigus vulgaris.

**Table 2:** Demographic and clinical characteristics of the study population (*n* = 10).

Variable	*n* (%)/Mean ± SD	Median (IQR)
Age (years)	68.4 ± 5.9	—
Female sex	6 (60%)	—
Male sex	4 (40%)	—
Disease duration (years)	—	3.2 (1.8–5.4)
Comorbidities	8 (80%)	—
Hypertension	6 (60%)	—
Type 2 diabetes	3 (30%)	—
Treatment type	—	—
Systemic corticosteroids	7 (70%)	—
Rituximab	2 (20%)	—
Combination therapy	1 (10%)	—

Legend: Data are presented as *n* (%) for categorical variables and as mean ± standard deviation (SD) or median (interquartile range, IQR) for continuous variables. Note: The total number of individual comorbidities exceeds the number of affected patients because some participants presented more than one concomitant condition (e.g., hypertension + type 2 diabetes).

**Table 3:** Multiple linear regression models predicting OP-QoLQ and DLQI total scores (*n* = 10).

Predictor	β (OP-QoLQ)	*p*-Value	β (DLQI)	*p*-Value
ABSIS total score	0.68	0.009	0.62	0.014
Disease duration	0.31	0.041	0.28	0.048
Age	0.12	0.276	0.09	0.314
Sex (female)	0.08	0.341	0.07	0.372

Legend: β = standardized regression coefficient. Models include ABSIS total score, disease duration, age, and sex as predictors. Adjusted R^2^ values were 0.62 for the OP-QoLQ model and 0.58 for the DLQI model, indicating substantial explained variance. Statistically significant predictors (*p* < 0.05) are shown in bold. Interpretation: Oral disease severity (ABSIS total score) was the strongest and most consistent predictor of QoL impairment. Longer disease duration further worsened QoL, reflecting cumulative functional and psychosocial burden. Age and sex had no significant effect, highlighting the dominance of disease-related over demographic factors in this older adult cohort.

## Data Availability

The data presented in this study are available on reasonable request from the corresponding author. The data are not publicly available due to privacy and ethical restrictions.

## Supplementary Materials

The following supporting information can be downloaded at: https://www.mdpi.com/article/10.3390/healthcare13222843/s1, Table S1: Study-Specific OP-QoLQ (items, response options, scoring, and domain structure).

## Abbreviations

The following abbreviations are used in this manuscript:

ABSIS	Autoimmune Bullous Skin Disorder Intensity Score
DIF	Direct Immunofluorescence
DLQI	Dermatology Life Quality Index
Dsg1	Desmoglein 1
Dsg3	Desmoglein 3
ERN-Skin	European Reference Network on Rare and Complex Skin Diseases
OHRQoL	Oral Health–Related Quality of Life
OP-QoLQ	Oral Pemphigus–Specific Quality of Life Questionnaire
PROs	Patient-reported outcomes
PV	Pemphigus vulgaris
QoL	Quality of Life
