# Supplementary material for: Patient-Reported Outcomes on Quality of Life in Older Adults with Oral Pemphigus
Source: Healthcare (Basel). 2025 Nov 9;13(22):2843. doi: 10.3390/healthcare13222843 (PMC12652617; doi:10.3390/healthcare13222843)
Supplement: Supplementary file 1 [file healthcare-13-02843-s001.zip › healthcare-3959913-supplementary.pdf]

Table S1. Study-Specific Oral Pemphigus QoL Questionnaire (OP-QoLQ, adapted): Items, Response Options, Scoring, and Domains

Recall period: last 2 weeks
Response scale (all items): 0 = Never, 1 = Rarely, 2 = Sometimes, 3 = Often, 4 = Very often
Direction: Higher scores indicate worse quality of life.

#	Item (patient-facing wording)	Domain
1	I experienced mouth pain or burning.	Physical symptoms
2	I noticed bleeding from oral sores/erosions.	Physical symptoms
3	My mouth was sensitive to spicy or acidic foods.	Physical symptoms
4	I had difficulty chewing solid or hard foods.	Functional limitations
5	I had difficulty swallowing because of mouth sores.	Functional limitations
6	I found it difficult to brush teeth or keep my mouth clean.	Functional limitations
7	I felt embarrassed about the appearance or symptoms in my mouth.	Emotional well-being
8	I felt anxious or worried about disease flare-ups.	Emotional well-being
9	I felt frustrated or irritable because of my mouth symptoms.	Emotional well-being
10	I avoided eating with others or in public because of mouth problems.	Social participation
11	I had difficulty speaking clearly due to mouth discomfort.	Social participation
12	I avoided social interactions or conversations because of symptoms.	Social participation
13	I was bothered by treatment side effects (e.g., sleep problems, mood changes).	Treatment burden
14	The time and effort required for treatment/appointments were burdensome.	Treatment burden
15	I had difficulty accessing or adhering to treatment (e.g., costs, availability).	Treatment burden

Scoring and Interpretation

Item scoring: 0–4 per item.

Domain subscales (0–12 each): sum the 3 items in each domain.

- Physical symptoms (Items 1–3)
- Functional limitations (Items 4–6)
- Emotional well-being (Items 7–9)
- Social participation (Items 10–12)
- Treatment burden (Items 13–15)

Total score (0–60): sum of all 15 items.

Suggested interpretation (pilot thresholds): 0–20 = mild impact; 21–40 = moderate impact; 41–60 = severe impact.

Handling Missing Data (pilot rules)

- Total score: if ≤2 items are missing, prorate: Total = (Sum of answered items / Number answered) × 15. If >2 items missing, do not report total (report domains only).

- Domain score: if 1 item is missing in a domain, use the mean of the other 2 items × 3 (rounded to one decimal). If ≥2 items missing in a domain, do not report that domain score.

- Always report the number of missing items alongside any prorated scores.

Administration Notes (geriatric-friendly)

Use ≥14-point font; allow neutral assistance for reading/marking responses when visual or motor limitations are present (without influencing answers). Ensure a quiet, private setting to minimize response bias.

Development Note

This study-specific, adapted instrument was derived from existing oral mucosal QoL frameworks and pemphigus literature, refined by an expert panel (dermatology, periodontology, geriatrics), and cognitively debriefed in older adults. No full psychometric validation (e.g., factor analysis, test–retest) has been completed; findings using this tool should be considered exploratory/hypothesis-generating.
